# Supplementary material for: Developmental kinetics and transcriptome dynamics of stem cell specification in the spermatogenic lineage
Source: Nat Commun. 2019 Jun 26;10:2787. doi: 10.1038/s41467-019-10596-0 (PMC6594958; doi:10.1038/s41467-019-10596-0)
Supplement: Supplementary file 1 — Supplementary Information [file 41467_2019_10596_MOESM1_ESM.pdf]

Supplementary Fig. 1

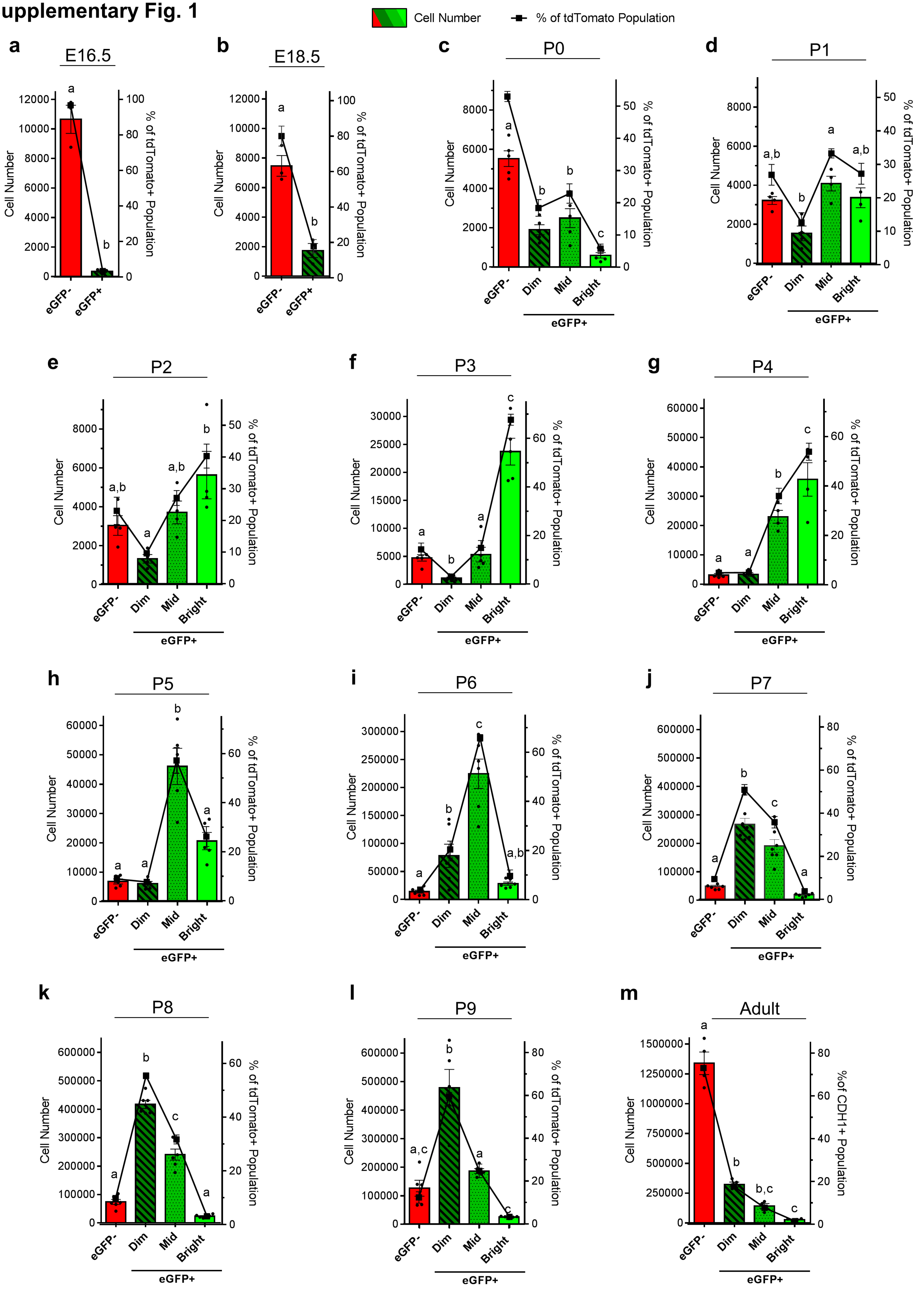

Supplementary Fig. 1: Quantification of each eGFP subtypes from E16.5 to adult. (**a-m**) Cell number (left axis and colored bars) and percent distribution (right axis, black squares, and black line) of eGFP populations from fetal, neonatal, and adult germ cell populations. Distributions in (a-l) are gated from all tdTomato+ germ cells and in (m) from all CDH1+ undifferentiated germ cells. Cell number data is presented with replicate values (circular black dots) and means with error bars representing SEM for n=3-7 biologically independent animals per age point (n=3 for E16.5 and E18.5; n=4 for P1, P2, P4, and adult; n=5 for P0, P3, P5, and P9; n=6 for P6 and P8; and n=7 for P7). Letters correspond to significantly different ($p<0.05$) groups as analyzed by t-Test (a, b) or one-way ANOVA (c-m). Data correspond to summary graphs in Fig. 2b and d.

Supplementary Fig. 2

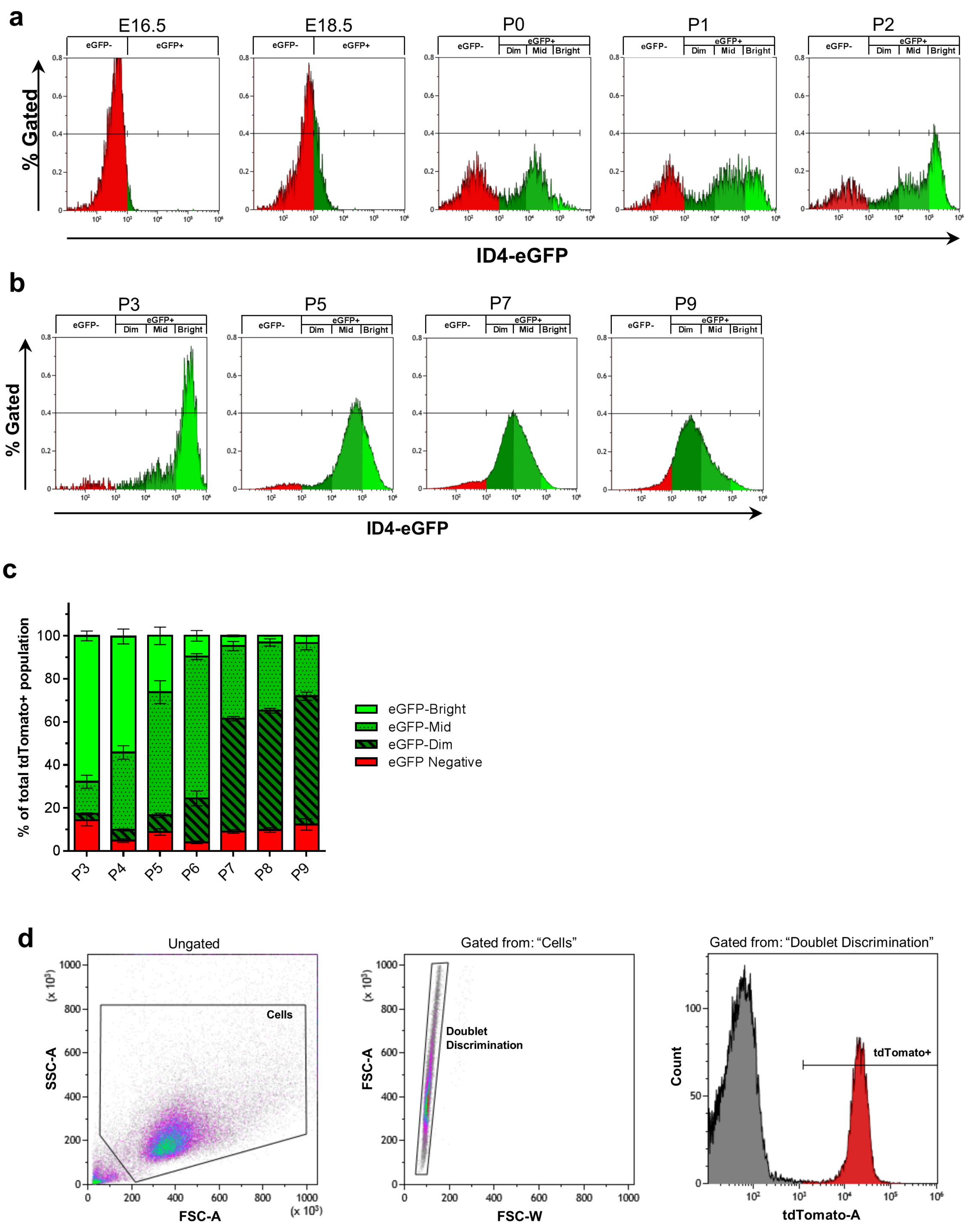

Supplementary Fig. 2: A gradient of eGFP expression forms during development. (a-b) Representative histograms of eGFP expression gated from all tdTomato+ germ cells during development. (**c**) Distribution of all eGFP populations, including eGFP negative germ cells, through developmental time. (d) Representative gating strategy for isolating tdTomato+ germ cells. Data in (c) are presented as means with error bars representing SEM for n=4-7 biologically independent animals per age point (n=4 for P4; n=5 for P3, P5, and P9; n=6 for P6 and P8; and n=7 for P7).

Supplementary Fig. 3

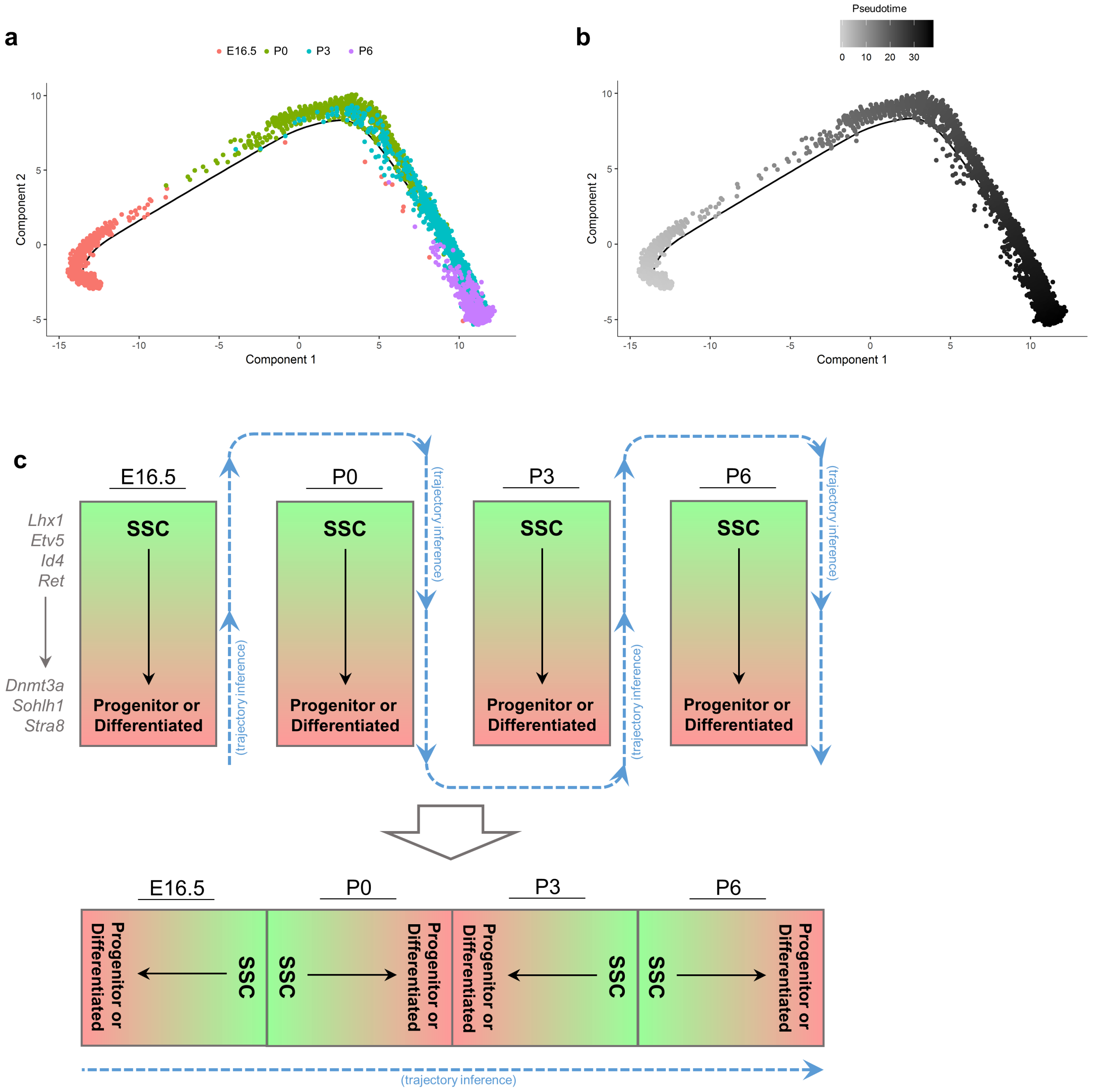

Supplementary Fig. 3: Trajectory inference algorithms are unable to resolve any unique germ cell lineages. (**a-b**) Representative Monocle trajectory analysis of all germ cells isolated from E16.5, P0, P3, and P6 testes (a) ordered in pseudotime (b). (**c**) Schematic of incorrect trajectory inference estimates due to dataset complexity.

Supplementary Fig. 4

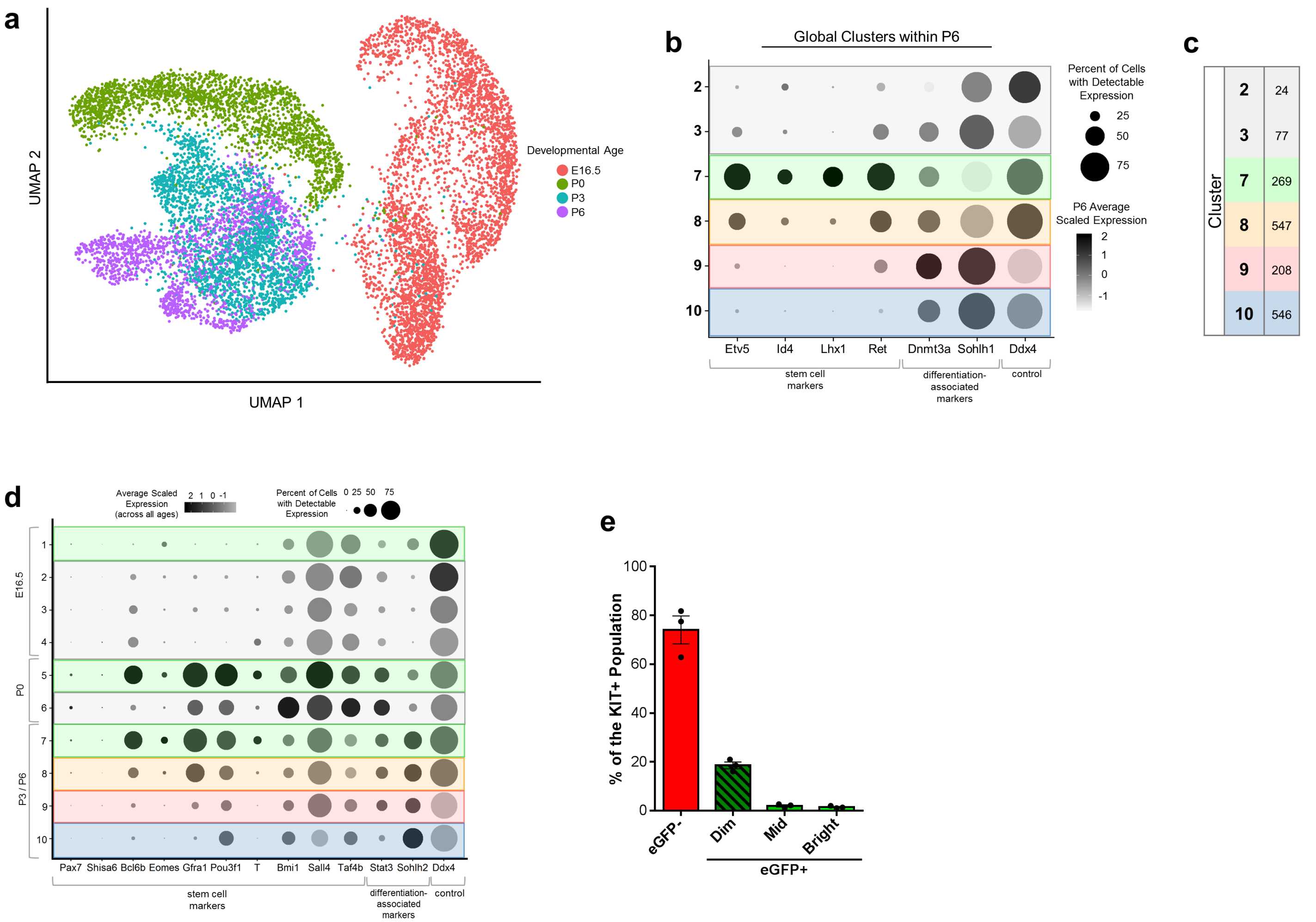

Supplementary Fig. 4: Marker gene profiling across developmental ages. (**a**) Uniform manifold approximation and projection (UMAP) representation of all four developmental ages assayed within the aggregate dataset. (**b-c**) Dotplot representation of marker gene expression (b) and cell distribution (c) for global clusters (continuation from Fig.4) present within the P6 dataset. (**d**) Additional SSC- and differentiation- associated markers that were excluded from cluster identification in Fig. 4f due to expression below the general thresholds described in Results (*Pax7* and *Shisa6*), scaled expression below 0 at E16.5 (*Bcl6b*, *Gfra1*, *Pou3f1*, and *T*), or unrestrictive expression among clusters (*Bmi1*, *Sall4*, *Taf4b, Stat3,* and *Sohlh2*). Dotplot representations in (b, d) indicate the average scaled gene expression of marker genes within P6 (b) or within all ages (d) (color gradient) and the percentage of cells with detectable expression for each marker (dot radius). scRNA-seq data from (a-d) are representative of a total of 10,140 cells from n=8 biologically independent animals. (**e**) FCA of KIT expression from eGFP germ cell subsets at P3. Data are presented as means with error bars representing SEM and black dots representing individual data points for the percentage of KIT+ cells for each eGFP subset. Data in (e) were collected from n=3 biologically independent animals.

Supplementary Fig. 5

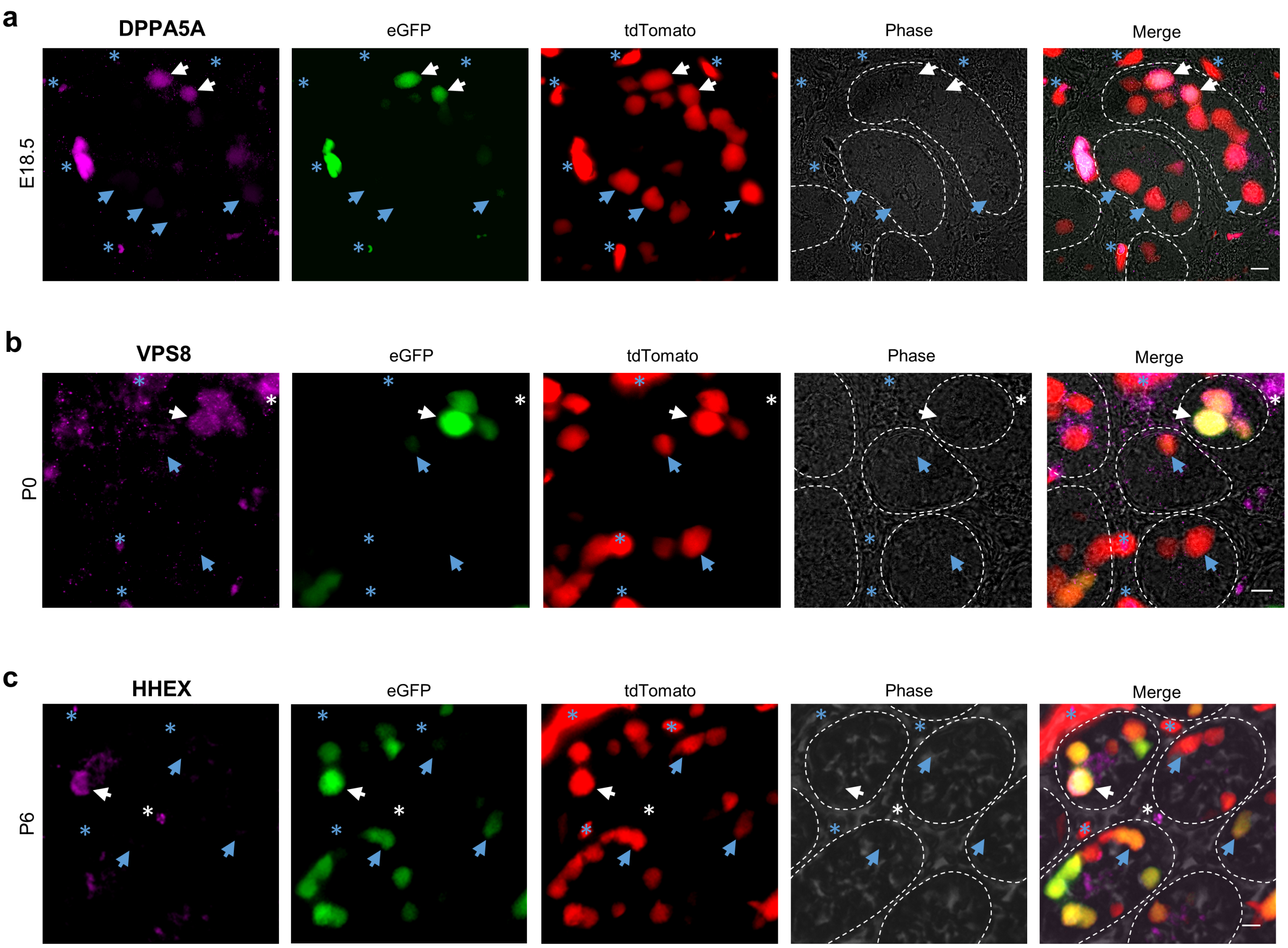

Supplementary Fig. 5: Additional validation of novel markers of SSC fate specification identified by scRNA-seq clustering. (**a-c**) Continued from Fig. 5, wider field images are presented that are representative of additional biological replicates for immunofluorescent staining for DPPA5A (a), VPS8 (b), and HHEX (c) proteins that were identified as differentially expressed at the transcript level in germ cell clusters 1, 5, and 7 of E18.5, P0, and P6 testes by scRNA-seq, respectively. Immunostaining of target proteins is paired with ID4-eGFP and tdTomato fluorescence in germ cells. White arrows indicate germ cells that are DPPA5A+ and ID4-eGFP+ at E18.5, VPS8+ and ID4-eGFP^{Bright} at P0, or HHEX+ and ID4-eGFP^{Bright} at P6. Blue arrows indicate germ cells that have low to undetectable staining for the selected marker and ID4-eGFP. Blue asterisks in (a-c) denote autofluorescence within the vasculature of testis cross-sections, and white asterisks in (b-c) denote interstitial cells (i.e. eGFP-/tdTomato-) that are either VPS8+ or HHEX+. Seminiferous tubule borders are approximated with white dotted lines based on phase images. Scale bars, 10 µm. Images are representative of ≥3 cross-sections imaged from n=2 biologically independent animals for each developmental age point.

Supplementary Fig. 6

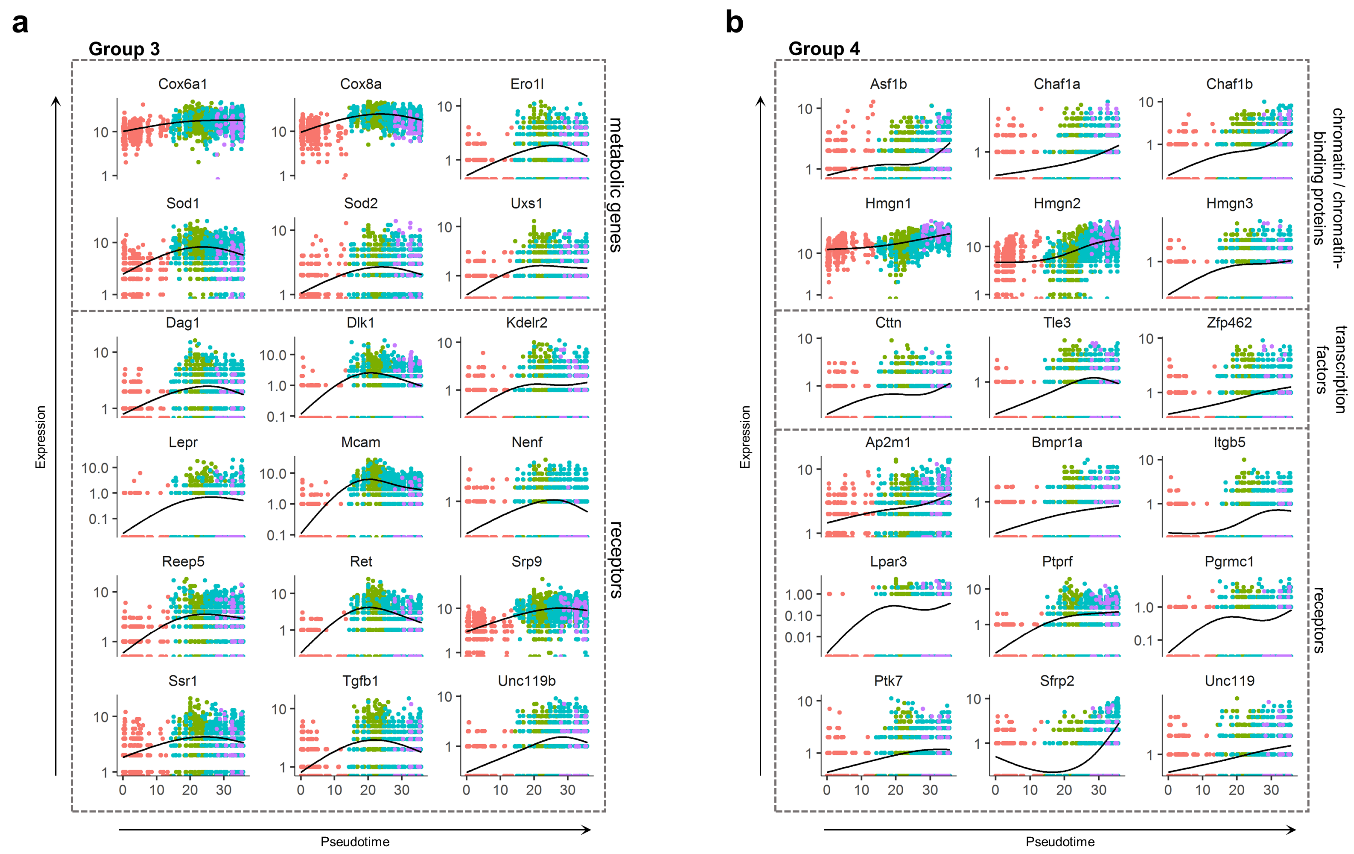

Supplementary Fig. 6: Gene families differentially expressed through pseudotime. (a-b) Select gene families upregulated mid-trajectory in group 3 (a) and upregulated late along the trajectory in group 4 (b) from SSC-enriched clusters. Data are a continuation of Fig. 6.
