# Supplementary material for: Developmental kinetics and transcriptome dynamics of stem cell specification in the spermatogenic lineage
Source: Nat Commun. 2019 Jun 26;10:2787. doi: 10.1038/s41467-019-10596-0 (PMC6594958; doi:10.1038/s41467-019-10596-0)
Supplement: Supplementary file 3 — Description of Additional Supplementary Files [file 41467_2019_10596_MOESM3_ESM.pdf]

Description of Additional Supplementary Files

File Name: Supplementary Data 1
Description: Differentially expressed genes among graph-based clusters. Included are the top ten differentially expressed genes from each of ten clusters depicted in the heatmap of Fig. 4c in order from top to bottom. Data are presented as the average log fold-change in expression between cells of the identified cluster and the remaining cells of the dataset. Also, the percentage of cells within and outside each designated cluster with detectable expression of each marker gene are reported. Adjusted p-values are given and p-values of 0 represent p<1.0x10-301.

File Name: Supplementary Data 2
Description: Genes differentially expressed along the SSC trajectory. A complete list of genes that are differentially expressed (p<0.01) as a function of pseudotime along the SSC trajectory and divided into four groups as presented in Fig. 4b. Group number and gene name are identified.
