# Supplementary material for: Developmental kinetics and transcriptome dynamics of stem cell specification in the spermatogenic lineage
Source: Nat Commun. 2019 Jun 26;10:2787. doi: 10.1038/s41467-019-10596-0 (PMC6594958; doi:10.1038/s41467-019-10596-0)
Supplement: Supplementary file 6 — Reporting Summary [file 41467_2019_10596_MOESM6_ESM.pdf]

nature research

Corresponding author(s): Jon Oatley

Reporting Summary

Nature Research wishes to improve the reproducibility of the work that we publish. This form provides structure for consistency and transparency in reporting. For further information on Nature Research policies, see Authors & Referees and the Editorial Policy Checklist.

Statistical parameters

When statistical analyses are reported, confirm that the following items are present in the relevant location (e.g. figure legend, table legend, main text, or Methods section).

n/a	Confirmed	
☐	☒	The exact sample size (*n*) for each experimental group/condition, given as a discrete number and unit of measurement
☐	☒	An indication of whether measurements were taken from distinct samples or whether the same sample was measured repeatedly
☐	☒	The statistical test(s) used AND whether they are one- or two-sided<br>*Only common tests should be described solely by name; describe more complex techniques in the Methods section.*
☒	☐	A description of all covariates tested
☐	☒	A description of any assumptions or corrections, such as tests of normality and adjustment for multiple comparisons
☐	☒	A full description of the statistics including central tendency (e.g. means) or other basic estimates (e.g. regression coefficient) AND variation (e.g. standard deviation) or associated estimates of uncertainty (e.g. confidence intervals)
☐	☒	For null hypothesis testing, the test statistic (e.g. *F*, *t*, *r*) with confidence intervals, effect sizes, degrees of freedom and *P* value noted<br>*Give P values as exact values whenever suitable.*
☒	☐	For Bayesian analysis, information on the choice of priors and Markov chain Monte Carlo settings
☐	☒	For hierarchical and complex designs, identification of the appropriate level for tests and full reporting of outcomes
☒	☐	Estimates of effect sizes (e.g. Cohen's *d*, Pearson's *r*), indicating how they were calculated
☐	☒	Clearly defined error bars<br>*State explicitly what error bars represent (e.g. SD, SE, CI)*

Our web collection on statistics for biologists may be useful.

Software and code

Policy information about availability of computer code

Data collection	Cell number, eGFP distribution, and cell cycle data was collected using Attune Nxt Software (v2.7.873). 3D confocal images were captured using Leica LasX (v3.3.0).
Data analysis	Statistical analysis of all cell number, eGFP distribution, cell cycle, and nest quantifications was performed using GraphPad Prism 6 software (v6.07). Cell number, eGFP distribution, and cell cycle data was analyzed with Kaluza Software (v 1.5a). 3D confocal images were quantified in ImageJ (v1.51t) with the 3D ROI Manager plugin. Using R software (v3.4.4), object distances were then used to perform single-linkage hierarchical clustering (hclust function) with a cutoff distance of 10 µm. For single-cell RNA-seq (scRNA-seq), raw sequencing reads were demultiplexed and aligned to the mouse genome using the 10X Genomics Cell Ranger pipeline (v2.1.0). scRNA-seq data was analyzed using R software (v3.4.4) with Seurat (v2.3.2) and Monocle (v2.6.4) packages. Gene Ontology was performed with DAVID (v6.8) or PANTHER (v13.1) software.

For manuscripts utilizing custom algorithms or software that are central to the research but not yet described in published literature, software must be made available to editors/reviewers upon request. We strongly encourage code deposition in a community repository (e.g. GitHub). See the Nature Research guidelines for submitting code & software for further information.

Data

Policy information about availability of data

All manuscripts must include a data availability statement. This statement should provide the following information, where applicable:

- Accession codes, unique identifiers, or web links for publicly available datasets
- A list of figures that have associated raw data
- A description of any restrictions on data availability

Transcriptome (scRNA-seq) data generated and analyzed during the present study are available from the GEO database (accession number GSE124904).

Field-specific reporting

Please select the best fit for your research. If you are not sure, read the appropriate sections before making your selection.

☒ Life sciences ☐ Behavioural & social sciences ☐ Ecological, evolutionary & environmental sciences

For a reference copy of the document with all sections, see nature.com/authors/policies/ReportingSummary-flat.pdf

Life sciences study design

All studies must disclose on these points even when the disclosure is negative.

Sample size	Each sample or experimental/biological replicate (n) represented one animal or a single testis from one animal (as noted in the text).
Data exclusions	No data was excluded from this study.
Replication	Reproducibility was ensured by including at least three biological replicates for each data point collected.
Randomization	Litters were selected at random for collection of each biological replicate (being one animal).
Blinding	Blinding was not necessary for this study because selection of animals for collection was entirely random.

Reporting for specific materials, systems and methods

Materials & experimental systems

n/a	Involved in the study	
☒	☐	Unique biological materials
☐	☒	Antibodies
☒	☐	Eukaryotic cell lines
☒	☐	Palaeontology
☐	☒	Animals and other organisms
☒	☐	Human research participants

Methods

n/a	Involved in the study	
☒	☐	ChIP-seq
☐	☒	Flow cytometry
☒	☐	MRI-based neuroimaging

Antibodies

Antibodies used	Anti-CDH1 (Biolegend, clone DECMA-1, catalog no. 147307, diluted 1:200)<br>Anti-Kit (Abcam, clone 2B8, catalog no. ab25495, diluted of 1:100)<br>Anti-DPPA5A (ThermoFisher, catalog no. PA5-48042, lot UB2715024A, diluted 1:20-1:40)<br>Anti-VPS8 (ThermoFisher, catalog no. PA5-57855, lot UB2715146A, diluted 1:50-1:100)<br>Anti-HHEX (ThermoFisher, catalog no. PA5-19625, lot UB2714724, diluted 1:50-1:100)
Validation	Antibodies were validated by the manufacturers.

Animals and other organisms

Policy information about studies involving animals; ARRIVE guidelines recommended for reporting animal research

Laboratory animals	Laboratory mice (Mus musculus) used in this study were of mixed 129/C57B6 background. Fetal (age indicated), neonatal (age

Laboratory animals	indicated), and adult (>P56) males were used for experimental analysis. All protocols were approved by the Washington State University Animal Care and Use Committee.
Wild animals	No wild animals were used.
Field-collected samples	No field-collected samples were used.

Flow Cytometry

Plots

Confirm that:

☒ The axis labels state the marker and fluorochrome used (e.g. CD4-FITC).

☒ The axis scales are clearly visible. Include numbers along axes only for bottom left plot of group (a 'group' is an analysis of identical markers).

☒ All plots are contour plots with outliers or pseudocolor plots.

☒ A numerical value for number of cells or percentage (with statistics) is provided.

Methodology

Sample preparation	Single cell suspensions were generated from isolated embryonic and neonatal testes by trypsin/EDTA digestion. Briefly, detunicated testes were incubated in a solution of 0.25% trypsin/EDTA (Thermo Fisher Scientific) and 2 mg/mL deoxyribonuclease I (Sigma-Aldrich, Inc.) for 10 min at 37C with gentle agitation. Trypsin digest was quenched with 10% fetal bovine serum (FBS) before cells were washed and resuspended in a solution of 1% FBS, 10 mM Hepes, 1 mM sodium pyruvate, 1 mg/mL glucose, 100 units/mL penicillin, and 100 g/mL streptomycin in PBS (Thermo Fisher Scientific or Sigma-Aldrich, Inc.). For adult mice, testes were first incubated in 1 mg/mL collagenase type IV (Thermo Fisher Scientific) at 37°C for 10 min with gentle agitation to disperse seminiferous tubules, rinsed three times with HBSS on ice to remove interstitial cells, and single cell suspensions generated by digestion with trypsin/EDTA solution. Because the Id4-eGfp transgene is expressed by pachytene spermatocytes, as well as SSCs, testis suspensions from adult Id4-eGFP males were incubated with an antibody recognizing the undifferentiated spermatogonial cell surface marker CDH1 (E-Cadherin) 74 (Biolegend, clone DECMA-1, catalog no. 147307) at a dilution of 1:200 for 30 min on ice and then gently washed three times before analysis. For KIT staining, cell suspensions were incubated with a fluorophore-conjugated antibody recognizing KIT (Abcam, clone 2B8, catalog no. ab25495) at a dilution of 1:100 for 30 min on ice and then gently washed three times before analysis.
Instrument	Cell suspensions were analyzed with an Attune Nxt Flow Cytometer (Thermo Fisher Scientific, Attune Nxt Software v2.7.873).
Software	Data was processed with Kaluza Analysis Software (Beckman Coulter, Inc., v1.5a).
Cell population abundance	Details regarding the fractions of each population based on eGFP intensity and/or cell cycle status are reported in the manuscript data.
Gating strategy	Background fluorescence was determined from unstained control testis suspensions of equivalent age. Single stained fluorescent controls were used for calculating compensation. After doublet discrimination, germ cells were gated from all tdTomato+ cells. For each sample, eGFP fluorescent signal was equally divided into thirds to establish Bright, Mid, and Dim designations, as described previously 24.

☒ Tick this box to confirm that a figure exemplifying the gating strategy is provided in the Supplementary Information.
